# From Concept to Representation: Modeling Driving Capability and Task Demand with a Multimodal Large Language Model

**DOI:** 10.3390/s25185805

**Published:** 2025-09-17

**Authors:** Haoran Zhou, Alexander Carballo, Keisuke Fujii, Kazuya Takeda

**Affiliations:** 1 Graduate School of Informatics, Nagoya University, Furo-cho, Chikusa-ku, Nagoya 464-8603, Japan; fujii@i.nagoya-u.ac.jp (K.F.); kazuya.takeda@nagoya-u.jp (K.T.); 2 Graduate School of Engineering, Gifu University, 1-1 Yanagido, Gifu City 501-1193, Japan; alex@gifu-u.ac.jp; 3 Institute of Innovation for Future Society, Nagoya University, Furo-cho, Chikusa-ku, Nagoya 464-8601, Japan; 4 Tier IV Inc., Nagoya University Open Innovation Center, 1-3, Mei-eki 1-Chome, Nakamura-ku, Nagoya 450-6610, Japan; 5 Institute of Physical and Chemical Research (RIKEN) Center for Advanced Intelligence Project, 1-5, Yamadaoka, Suita 565-0871, Japan

**Keywords:** task–capability interface model, representation learning, multimodal large language model, driving capability, task demand, task difficulty

## Abstract

Driving safety hinges on the dynamic interplay between task demand and driving capability, yet these concepts lack a unified, quantifiable formulation. In this work, we present a framework based on a multimodal large language model that transforms heterogeneous driving signals—scene images, maneuver descriptions, control inputs, and surrounding traffic states—into low-dimensional embeddings of task demand and driving capability. By projecting both embeddings into a shared latent space, the framework yields an interpretable measurement of task difficulty that alerts to capability shortfalls before unsafe behavior arises. Built upon a customized BLIP 2 backbone and fine-tuned on diverse simulated driving scenarios, the model respects consistency within tasks, captures impairment-related capability degradation, and can transfer to real-world motorway data without additional training. These findings endorse the framework as a concise yet effective step toward proactive, explainable risk assessment in intelligent vehicles.

## 1. Introduction

Traffic accidents remain a critical social issue. According to a recent report from the World Health Organization (WHO), road accidents cause more than 1.2 million fatalities per year globally [1], and human error is estimated to contribute to roughly 94% of accidents [2]. Various active safety systems have been developed for decades—ranging from fundamental technologies such as Anti-lock Braking Systems (ABS) and Electronic Stability Control (ESC), to Advanced Driver-Assistance Systems (ADAS) integrating functions like Adaptive Cruise Control (ACC), Autonomous Emergency Braking (AEB), and Lane Keeping Assist (LKA), and more recently, to assisted and autonomous driving systems [3]—all aiming to reduce and finally prevent traffic accidents. However, even the latest automated driving technologies have not eliminated collisions. An accident occurred in 2019 in Delray Beach when a Tesla in Autopilot mode collided with a truck, resulting in the death of the driver [4]. In 2023, a Cruise autonomous taxi in San Francisco struck a pedestrian and dragged her for approximately 20 feet [5]. These cases illustrate that state-of-the-art driving systems, much like human drivers, still encounter situations beyond their capabilities, leading to accidents.

**Figure 1:**
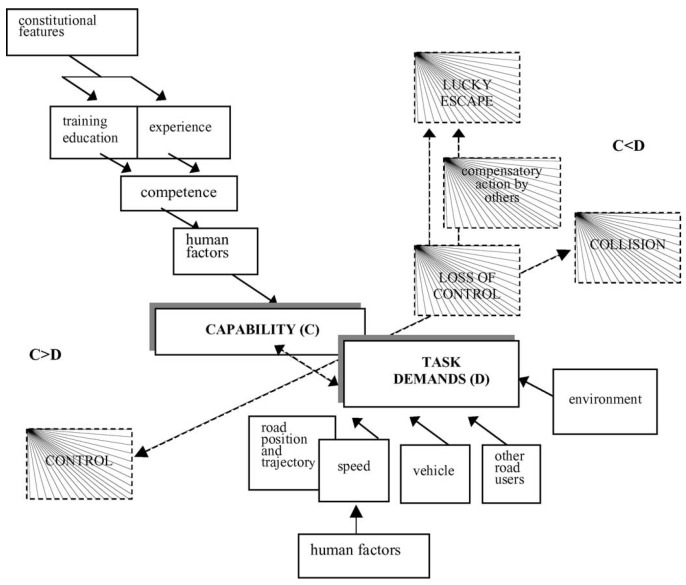
Illustration of the TCI model. Reproduce from [7], © 2005 by Elsevier, with permission.

Previous research efforts [6,7] have pointed out a fundamental cause among these crashes: a misalignment between driving capability and task demand. As shown in Figure 1, in Fuller’s Task–Capability Interface (TCI) model, safe driving is maintained only when the driving capability meets or exceeds the momentary demands of the driving task. If the required task demand exceeds the capability of the human driver or the autonomous system, the risk of an accident increases dramatically, further leading to a collision if unfortunately. In practice, this capability–demand misalignment can occur due to various factors: a human driver might be fatigued, distracted, or unskilled in a challenging scenario, or an autonomous driving system might face an edge case outside its operational design domain.

Recognizing this misalignment in time or even in advance could enable preventive interventions to mitigate the associated risks. For example, alerting a distracted driver to refocus, engaging emergency braking, or prompting a Level-3 automated vehicle to hand control back to a more capable driver. Unfortunately, accomplishing this in practice has been challenging, in part because driving capability and task demand are not formally defined or directly measurable in existing frameworks. These concepts are multifaceted and dynamic, influenced by vehicle state, environmental complexity, and human factors, and there is no universally accepted quantitative model for these terms [8]. As a result, current driver-assistance systems lack a rigorous basis to evaluate whether a driver (human or AI) can cope with an upcoming situation, making further intervention difficult to justify or execute.

Multimodal Large Language Models (MLLMs) offer a promising new avenue to address this challenge. Recent breakthroughs in artificial intelligence have produced large models that learn from massive amounts of multimodal data, enabling an unprecedented level of general scene understanding and reasoning. In the driving domain, such a model could analyze traffic scenes (vehicles, pedestrians, road signs, weather conditions, etc.) while also considering the descriptive context (traffic rules, learned common sense about how scenarios typically unfold) [9]. Leveraging these advantages, this work proposes a novel MLLM-based approach to formally model driving capability and task demand. By harnessing the generalization power and multimodal perception of MLLM, we aim to bridge the definitional gap and develop a data-driven framework for identifying driving capability shortfalls before they lead to accidents.

The main contributions of this paper can be summarized as follows.

- We adapt the architecture of BLIP-2 [10] with a novel custom design aligned with the TCI model, enabling formal modeling of task demand and driving capability. This tailored architecture provides a principled framework for capturing and analyzing alignments in dynamic driving scenarios.
- We propose a new method to explicitly distill low-dimensional semantic vectors for driving capability and task demand. By imposing a dimensionality constraint on the MLLM’s output and integrating a multi-label contrastive learning objective, our approach yields concise yet expressive representations that characterize the driving capability and the task demand within a shared latent space.
- We design a comparator module to measure the distance between capability vectors and between capability and demand vectors. This enables the effective detection of mismatches between driving capability and task demand, thereby facilitating early intervention and risk awareness. Validation and analysis across multiple datasets demonstrate the practical feasibility and interpretability of the proposed method.

The remainder of this paper is organized as follows. In Section 2, we provide a review of the literature of prior work relevant to this study. Section 3 details the research problem addressed in this study and the methodologies employed. The dataset we constructed to support this work is introduced in Section 4. In Section 5, we present the evaluation, analysis, and ablation study of the proposed method. Following in Section 6, we discuss the key findings, challenges encountered, and potential future research directions. Finally, Section 7 concludes this paper.

## 2. Related Works

### 2.1. Task–Capability Interface Model

The Task–Capability Interface (TCI) model, which was proposed by Fuller in 2000, conceptualizes driving as the “management of task difficulty” where task difficulty correlates with the probability of risk occurrence [6]. Within this context, task difficulty is determined by two key components: task demand and driving capability. The task difficulty arises from the dynamic discrepancy between these two components [11]. When driving capability significantly exceeds task demand, the task is perceived as easy; conversely, when task demand approaches or exceeds the driving capability, the task is considered difficult, resulting in the driver’s inability to maintain complete control of the vehicle, which may consequently result in an accident [7]. In the framework of the TCI model, task demand refers to the objective complexity of the current driving scenario, primarily influenced by the driving environment, surrounding vehicles, other traffic participants, and the driver’s intentioned maneuvers such as trajectory and speed selection. In comparison, driving capability denotes the momentary delivery of competence by the driver during the process. Here, competence broadly encompasses driving knowledge and skills such as vehicle control, road observation, and anticipation.

Subsequently, the TCI model has been employed as a theoretical tool in studies of driving behaviors in various scenarios. From the perspective of driving capability, different groups of drivers (e.g., drivers differing in driving experience [12,13], age [12,14,15], or gender [13]), or drivers operating in different physical [15,16,17,18] or mental [19,20,21] states, exhibit different driving capabilities, which consequently lead to variations in driving behaviors. Regarding task demand, attention focuses primarily on how the behavior of similar drivers varies when faced with different speeds [19,22] and levels of environmental complexity [15,23]. The TCI model provides researchers with a robust theoretical framework for better understanding and explaining the causal relationships underlying driver behaviors.

When employing the TCI model to infer behaviors and evaluate driving performance, however, the situation becomes somewhat more complex, primarily due to the lack of concretely specifying its internal mechanisms [24,25]. Driving capability and task demand lack standardized numeric definitions and, since they operate on different conceptual dimensions, direct quantitative comparisons are not feasible. Moreover, since inherent driving competence can only set an upper limit on the driving ability delivered, actual driving capability must be assessed dynamically during driving [26]. These factors further complicate the functional implementation of the TCI model. Although some studies attempt to regard task difficulty as driving risk [27] or driver fitness [28] in order to circumvent the need for direct use of driving capability and task demand, it is believed that explicit modeling of driving capability and task demand has significant potential to improve real-time safety interventions and adaptive driving assistance.

### 2.2. Impaired Driving

Impaired driving refers to the driving operations of a driver whose ability is compromised. The impairment may manifest itself as reduced reaction time, distracted attention, improper decision making, or decreased motor coordination [1]. Within the framework of the TCI model, impaired driving can be understood as a state in which temporary human factors cause the delivered driving capability to fall below the expectation, resulting in a mismatch with task demand and consequently a risk of losing control and accident.

Among all forms of impaired driving, the most common is Driving Under the Influence (DUI) of alcohol or drugs, especially under the influence of alcohol [29]. For example, in Japan, DUI-related accidents accounted for 5.5% of all personal injury traffic accidents in 2017 [30]. DUI is manifested through various observable signals such as body movements [31,32,33] and facial expressions [34,35,36], but the primary impact lies in the driver’s operation of vehicle controls, such as the input of the steering and pedals [37]. For example, intoxicated drivers tend to make more frequent steering corrections compared to their normal state [38]. Furthermore, studies have shown that alcohol consumption can alter a driver’s personal driving style [39], further affecting the decisions and actual capability delivered while driving.

Other frequently observed forms include drowsiness, distraction, and extreme emotions. Drowsiness is considered to produce effects similar to those of alcohol [40], such as reducing the ability to control steering and braking and increasing the variability of driving speed [41,42,43]. Distraction, either due to events within [44,45] or outside [46,47] the vehicle, leads to allocation of the attentional resource to a secondary activity, thereby increasing the probability of missing critical events during driving [48]. Extreme emotional states such as anger, anxiety, or sadness can affect the judgment of a driver and result in the adoption of more aggressive or erratic driving styles, such as sudden acceleration, tailgating, or risky overtaking maneuvers [49,50,51].

In summary, while the causes and manifestations of impaired driving may vary, a consistent feature is that the driver’s performance under impairment is, to some extent, degraded compared to their normal state. Under equivalent task demand, this degradation can be interpreted as a reduction in the driving capability delivered by the driver, resulting in a lower output relative to their unimpaired condition.

### 2.3. Multimodal Large Language Model in Driving

With recent advances in computational hardware, the implementation of Large Language Models (LLM) based on the Transformer architecture [52] has become feasible, as exemplified by models such as PaLM [53], LLaMA [54], GPT-4 [55], etc. These models, trained on massive amounts of internet data, can capture complex semantic patterns and perform at or beyond human levels in tasks such as language understanding, reasoning, and text generation [56,57,58]. An increasing number of applications [59,60,61] are now exploring the development of intelligent agents powered by LLMs to assist or even replace human involvement, including in the field of driving.

In driving scenarios, vehicles typically rely on a variety of sensors such as cameras, LiDARs, radars, and Controller Area Network (CAN) bus systems to perceive the surrounding environment and monitor internal states. Consequently, intelligent agents designed for driving must be LLMs that are capable of processing multimodal data, i.e., the Multimodal Large Language Model (MLLM) [62]. One of the most representative classes of such models is the Vision-Language Model (VLM), which takes natural language and images as input and performs tasks such as image captioning and visual question answering (VQA), such as CLIP [63], ViLBERT [64], BLIP-2 [10], and Flamingo [65]. In addition to these, audio [66,67] and pointcloud [68] are also frequently used modalities in driving applications.

MLLMs offer significant advantages in driving, particularly in their ability to fuse multiple modalities and generalize to novel, unseen situations [63,65,69]. Currently, research on applying MLLMs in driving focuses mainly on two core tasks: perception and planning/control. Discriminative perception models such as HiLMD [70] and Talk2BEV [71] utilize video and natural language input to generate visuo-linguistic responses related to driving, while generative models such as GAIA-1 [72] and UniSim [73] can anticipate environmental dynamics based on previous driving maneuvers. In the domain of planning and control, the goal extends beyond the understanding of the scenario: It involves using language instructions to recommend future waypoints to accomplish the current driving task [74,75], or even directly generating control signals to operate the vehicle [76,77]. Although still in the early stages, research into MLLMs in driving is rapidly gaining momentum, with more promising developments expected in the near future.

## 3. Methodology

### 3.1. Problem Statement

As shown in Figure 1, in the Task–Capability Interface (TCI) model, task demand is defined by the driving environment and the intended driving maneuvers, while driving capability is constrained by driver maximum driving competence and influenced by various human-related factors, reflected in actual driving behaviors. In the previous study, an important observation is that drivers in any case always attempt to dynamically allocate only the most appropriate level of capability in response to the specific driving context, to maintain perceived task difficulty at a preferred level [26]. And empirical evidence also suggests that the intended maneuvers of a driver and the maneuvers ultimately executed are, in practice, highly congruent. As a result, we define a unified input *X* as a pair of driving environment $X^{e}$ and driver behavior $X^{b}$:

(1) $$X=(X^{e},\;X^{b})$$

Based on this input, here two functions $f_{d}\left(\cdot \right)$, $f_{c}\left(\cdot \right)$ are defined to map the driving context to a latent representation to formally capture task demand and driving capability:

(2) $$d=f_{d}\left(X\right)$$

(3) $$c=f_{c}\left(X\right)$$

where $d,\;c\in R^{n}$ are semantic embeddings that lie in a comparable space, enabling the subsequent computation of task difficulty $\Delta_{d}$ as a function of the discrepancy between task demand d and driving capability c, formally defined as:

(4) $$\Delta_{d}=g\left(d,c\right)$$

where $\Delta_{d}\in R$ denotes the task difficulty, g(·,·) quantifies the discrepancy between demand and capability.

To obtain meaningful embeddings of demand and capability, the following properties are imposed as conceptual constraints or regularization objectives. These properties ensure that the obtained embeddings d and c align with intuitive and theoretical expectations:

**Property** **1** (Task Consistency). *Task-demand embeddings d and the corresponding delivered driving capability embeddings c should remain consistent in similar scenarios $X_{i}$, $X_{j}$, while differentiating clearly between semantically dissimilar scenarios $X_{i}$, $X_{k}$.*

(5) $$∥f_{d}\left(X_{i}\right)-f_{d}\left(X_{j}\right)∥\ll ∥f_{d}\left(X_{i}\right)-f_{d}\left(X_{k}\right)∥$$

(6) $$∥f_{c}\left(X_{i}\right)-f_{c}\left(X_{j}\right)∥\ll ∥f_{c}\left(X_{i}\right)-f_{c}\left(X_{k}\right)∥$$

**Property** **2** (Intra-task Capability Ordering). *For a given task $X_{i}^{e}$, the embeddings of the different delivered driving capabilities $c_{i}^{\left(j\right)}$ should be orderable, reflecting their relative adequacy in fulfilling the task demand.*

(7) $$c_{i}^{\left(1\right)}≺c_{i}^{\left(2\right)}≺\ldots ≺c_{i}^{\left(m\right)}$$

(8) $$c_{i}^{\left(m\right)}=f_{c}\left(\left(X_{i}^{e},X_{m}^{b}\right)\right)$$

**Property** **3** (Task–Capability Comparability). *For a given scenario $X_{i}$, the embeddings of task demand $d_{i}$ and the driving capability delivered $c_{i}$ should be comparable in a shared latent space. The comparison result should reflect the principle in the TCI model: task difficulty increases when the demand exceeds the capability, and remains low when the capability meets or exceeds the demand.*

(9) $$\Delta_{d}^{i}=g\left(d_{i},c_{i}\right)$$

(10) $$c_{i}\geq d_{i}\Rightarrow \Delta_{d}^{i}\approx 0,\;c_{i}<d_{i}\Rightarrow \Delta_{d}^{i}↑$$

### 3.2. Objective and Framework Overview

The goal of this research is to obtain descriptors that can explicitly represent driving capability and task demand, and to assess the task difficulty in the current driving situation by comparison between the two components. Specifically, the study consists of two main objectives:

1. Learning and modeling the functions $f_{d}\left(\cdot \right)$, $f_{c}\left(\cdot \right)$, g(·,·) introduced in Section 3.1.
2. Validating the success of the modeling by examining whether the properties described in Section 3.1 are satisfied.

**Figure 2:**
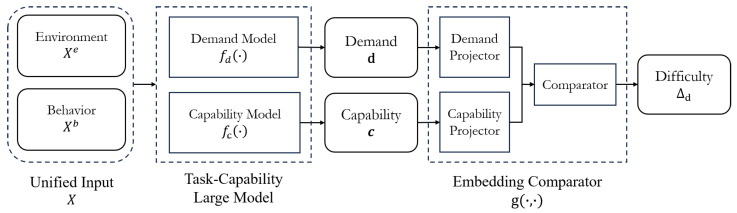
Overall structure of the proposed framework.

To this end, a novel framework is proposed in this study to model both task demand and driving capability through data-driven representation learning. The general structure of the framework is illustrated in Figure 2. The proposed framework unifies the driving environment and the driver behavior as the input, using a multimodal large language model (MLLM) to encode the input into latent embeddings of task demand and driving capability. These embeddings are fed into a lightweight comparator that projects both vectors into a shared, comparable space, yielding scalar scores that quantify their relative magnitudes. By contrasting the demand score with the capability score, the framework finally estimates the task difficulty for the current driving scenario.

### 3.3. Task–Capability Large Model

**Figure 3:**
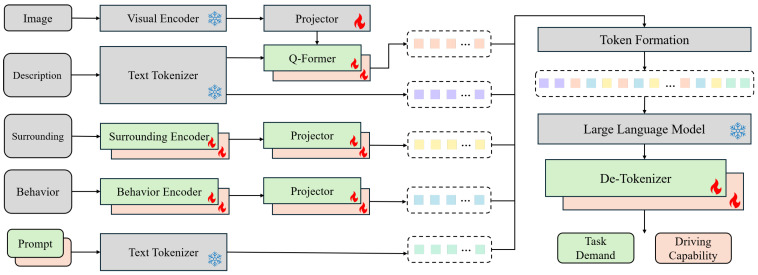
Network structure of the task–capability large model. A flame marks modules whose parameters are trainable, while a snowflake marks those kept frozen. Color coding denotes the pipeline in which each component is used: green for task demand, orange for driving capability, and gray for components shared by both.

In this study, we employ an MLLM-based task–capability large model to model the function $f_{d}\left(\cdot \right)$ and $f_{c}\left(\cdot \right)$ described in Section 3.1. The model takes a multimodal input of the driving environment and the driver behavior, and outputs latent embeddings for both task demand and driving capability. The architecture of the network is illustrated in Figure 3.

The task–capability large model is designed based on the BLIP-2 [10] architecture, utilizing a pre-trained visual encoder and a pre-trained large language model (LLM) with the corresponding text tokenizer to reduce the dependence on large-scale data and computational resources. In addition to scene image, natural language description of the current driving scenario, and task prompt also used in BLIP-2, the task–capability large model also incorporates modalities including driver behavior data (such as manipulations of the steering wheel and the accelerator or brake pedals) and surrounding vehicle information (such as position and speed). In the implementation, the surrounding encoder and the behavior encoder first employ a lightweight three-layer Multi-Layer Perceptron (MLP) to extract features from each frame. The resulting feature vectors are then partitioned into patches along the temporal dimension and integrated by a six-layer transformer encoder to capture spatio-temporal representations. Subsequently, a projector composed of fully connected layers aligns the output with the input dimension of the LLM. The capability and demand pipelines share an identical architecture; only certain modules differ in their parameter settings. Each modality is encoded into tokens and aligned with LLM’s input space by its respective encoder and projector, and the natural language description is used to query a subset of key visual tokens via a BLIP-2-style Q-former module.

**Figure 4:**
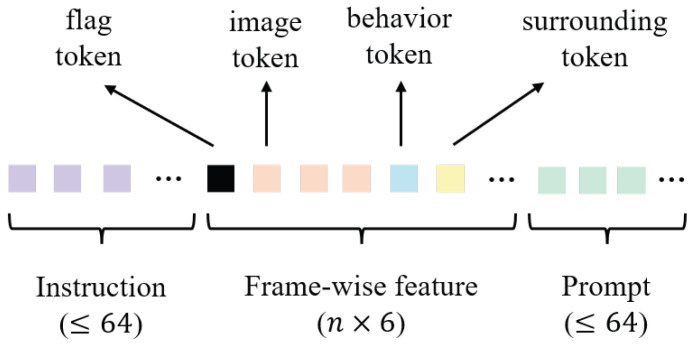
Illustration of the structure of the LLM input token series. Each color corresponds to a specific modality of tokens. *n* indicates how many image frames are included in the input.

Different modalities of tokens are subsequently formed for LLM input. The structure of the series of LLM input tokens and the token number for each part are shown in Figure 4. For each image frame in a data sample, the Q-Former selects four key representative tokens, concatenates them with a behavior token and a surrounding token of the corresponding frame, and a special flag token is prefixed to mark the beginning of the frame-wise feature sequence. Frame-wise feature sequences are first stitched together, after which the instruction tokens are added to the front and the prompt tokens to the end. The entire token sequence is then fed into the LLM to obtain hidden states, which are decoded by a three-layer MLP de-tokenizer into the corresponding representation embeddings.

To obtain meaningful representations and explicitly endow them with Property 1, we extend the previous Supervised Contrastive learning (SupCon) method [78] to train the model in a multi-label manner. In accordance with previous works [38,39], scene category and action category are used as supervisory labels for task demand, while driver ID and driver state are also employed to supervise driving capability. For each backpropagation step, a batch of normalized representations $Z=[z_{1},z_{2},\ldots ,z_{B}]$ is produced and a Boolean relation matrix $R\in {\left\{0,1\right\}}^{B\times B}$ is generated for each label, where $R_{ij}=1$ means sample *i* and *j* share the same attribute on this label. First, a normalized cosine similarity matrix *S* is calculated for every pair of samples in the batch. Each element $S_{ij}$ of the matrix is calculated as follows:

(11) $$S_{ij}=\frac{z_{i}^{⊤}z_{j}}{\tau }$$

τ is a temperature hyperparameter to scale the similarity. For every anchor *i*, a categorical distribution is computed over other samples in the batch, the logarithmic probability $\log p_{ij}$ is given by:

(12) $$\log p_{ij}=S_{ij}-\log\;\left(\sum_{k\neq i}e^{\;S_{ik}}\right),\;j\neq i$$

In addition, the supervised contrastive loss L is calculated as:

(13) $$L=-\frac{1}{\sum_{i,j}R_{ij}}\sum_{i,j}R_{ij}\;\log p_{ij}$$

With multiple relation matrices $R^{\left(k\right)}$, the final object $L_{\mathrm{total}}$ is:

(14) $$L_{\mathrm{total}}=\sum_{k}\lambda_{k}L\;\left(Z,R^{\left(k\right)}\right)$$

where $\lambda_{k}$ weighs different attributes. This loss encourages samples that share a larger number of labels to be closer in the embedding space while automatically generating negative pairs from the remaining samples in the batch.

### 3.4. Embedding Comparator

**Figure 5:**
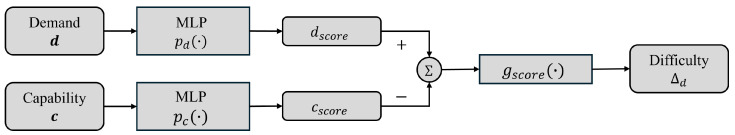
Network structure of the embedding comparator.

After obtaining the representations of task demand d and driving capability c, we further model g(·,·) with an embedding comparator. The structure of the embedding comparator is illustrated in Figure 5. Specifically, d and c are projected into a scalar space with the respective three-layer MLP to produce $d_{\mathrm{score}}$ and $c_{\mathrm{score}}$. The $d_{\mathrm{score}}$ and $c_{\mathrm{score}}$ are designed to enhance interpretability, which are obtained through unsupervised learning to represent the relative magnitudes of task demand and driving capability within a category. The difference between the scores is then calculated, and this difference is subsequently mapped to the final difficulty measure $\Delta_{d}$.

**Figure 6:**
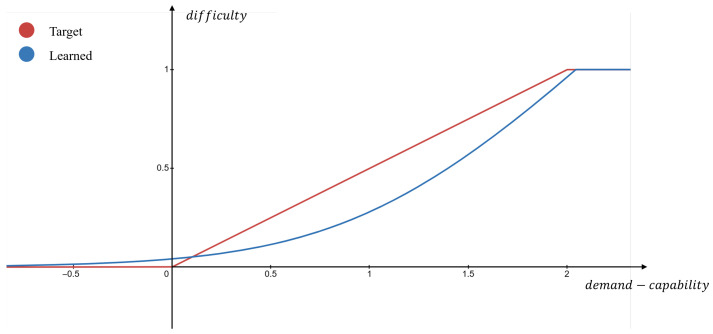
Illustration of the target (red) and the learned (blue) demand–capability comparison function.

As mentioned in Property 3 and illustrated in Figure 6, task difficulty should increase when demand exceeds capability and remain low when capability meets or exceeds demand. Consequently, a learnable function $g_{\mathrm{score}}\left(\cdot \right)$ is introduced within the embedding comparator to fit the target function, defined as follows:

(15) $$g_{\mathrm{score}}=\log\left(1+\alpha e^{\beta (d_{\mathrm{score}}-c_{\mathrm{score}})}\right)$$

where α and β are trainable parameters, α controls the growth scale, and β controls the growth rate. Compared with simple ReLU-like functions, the proposed function can more smoothly capture task difficulty when demand and capability are close, while producing a sharp increase in task difficulty when the gap is large, better reflecting how task difficulty is perceived in real driving scenarios. To exhibit Property 3, the predicted task difficulty $\Delta_{d}$ is compared with the ground-truth task difficulty $\Delta_{gt}$ to ensure a meaningful comparability, and the regression loss is computed as follows, where *N* denotes the number of samples:

(16) $$L_{\mathrm{reg}}=\frac{1}{N}\sum_{i=1}^{N}{\left(\Delta_{d}^{i}-\Delta_{gt}^{i}\right)}^{2}$$

To ensure that the extracted capability exhibits Property 2, we introduce the following assumption to impose an order relation on certain pairs of driving capabilities:

**Assumption** **1.** *For a given task, a driver always exhibits a higher driving capability in a normal state compared to an impaired state.*

Based on this assumption, we pair driving capability samples $\left[d_{\mathrm{normal}},d_{\mathrm{impaired}}\right]$ obtained from the same driver and the same scenario in different states, and employ a pairwise logistic loss $L_{\mathrm{pair}}$ [79], defined as:

(17) $$L_{\mathrm{pair}}=\sum_{\left(i,j\right)}\log\left(1+\exp\left(p_{d}\left(d_{\mathrm{impaired}}\right)-p_{d}\left(d_{\mathrm{normal}}\right)\right)\right)$$

This pairwise logistic loss $L_{\mathrm{pair}}$ automatically determines the appropriate margin of the data with respect to the ordering constraint, thereby enforcing an ordinal relationship among different capabilities. The total loss $L_{\mathrm{total}}$ for the embedding comparator is the sum of the regression loss $L_{\mathrm{reg}}$, the pairwise logistic loss $L_{\mathrm{pair}}$:

(18) $$L_{\mathrm{total}}=L_{\mathrm{reg}}+L_{\mathrm{pair}}$$

## 4. Dataset

### 4.1. Overview

**Figure 7:**
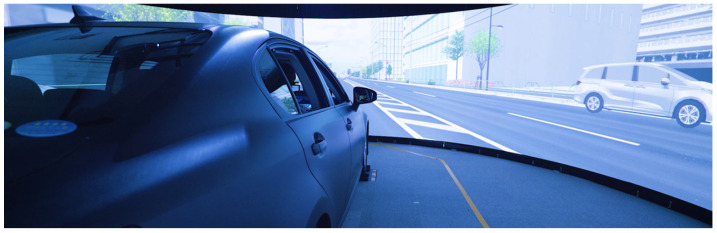
The driving simulator for collecting driving data. Reproduced from [38], distributed under the terms of the Creative Commons Attribution License (CC BY 4.0).

As discussed in Section 3.4, obtaining comparable representations of driving capability requires a dataset that contains multiple instances of driving behavior under similar tasks and admits a partial order of these instances with respect to the capability of the driver. To meet this requirement, we leverage a dataset which was built and described in detail in our previous works [38,39]. The dataset was constructed with the driving simulator provided by the Advanced Research and Innovation Center, DENSO CORPORATION, as illustrated in Figure 7. For each driving task, the same driver performed both normal and drunk sessions, enabling direct, task-consistent comparisons.

In this study, following the guidance of previous research [6,7,80], we investigated several representative driving tasks in urban scenarios. These tasks were segmented into approximately 32,000 samples, each 10 s long, totaling approximately 5300 min of driving data. Each data sample contains multimodal information, including scenario and driver metadata, natural language task descriptions, camera images, driver operation signals, and the behaviors of related surrounding traffic participants. This dataset was used for finetuning the model and for conducting part of the experimental validation.

### 4.2. Scenario Categorization

For both driving capability and task demand, the differences between scenarios are not merely quantitative but qualitative in nature. For example, during car-following on a straight road, the driver primarily needs to control the speed and maintain the distance using the accelerator and brake pedals, while at intersections involving turns, steering control becomes equally essential. As a result, categorizing the appropriate driving scenarios becomes essential.

In the dataset, a two-step procedure is adopted to categorize the scenarios. The first step involves segmentation based on the speed of the ego vehicle. In the TCI model, the task demand is positively correlated with the vehicle speed: when the speed is zero, the task demand is also considered zero [6]. In such cases, since no active driving effort is required, the scene does not contribute meaningful information to the driving ability and is therefore excluded from further analysis. Subsequently, the segmented driving clips were classified according to the geometry of the road (non-intersection, branch zone, merge zone, intersection) and vehicle behavior (going straight, lane change, turning) according to the criteria defined in [80]. In the dataset, it primarily considers vehicle behaviors of going straight and turning, and road structures of intersections and non-intersections (including both straight and curve).

**Figure 8:**
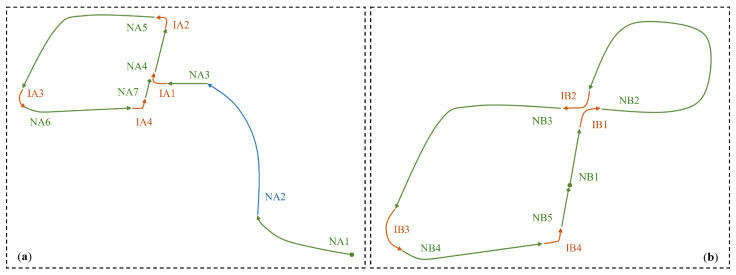
Illustration of the categorized scenarios: Circles and diamonds denote the start and end points, respectively; Arrows indicate the travel direction. Scenario categories are color-coded: green for non-intersection, blue for non-intersection (tunnel), and orange for intersections. Each scenario is represented using a code: NA for non-intersection in Route A; IB for intersection in Route B. (**a**) Route A—urban road with moderate traffic; (**b**) Route B—urban road with light traffic and pedestrian presence.

Figure 8 presents the result of the categorization of scenarios. In this study, two driving routes (Route A and Route B) are utilized, both constructed based on real urban traffic environments in Japan. Route A shown in Figure 8a features moderate traffic, with the ego vehicle primarily engaged in car following behavior. In comparison, Route B in Figure 8b has lower traffic density but includes several pedestrian-related events, such as crossing pedestrian crosswalks. The route shown in Figure 8a contains a tunnel segment which, due to its markedly different environment, is treated as a separate segment (NA2) in our analysis.

### 4.3. Data Modality

After obtaining the categorized scenarios, it is further segmented into samples of 10 s duration following the methodology of previous studies [38,39]. Each sample comprises various modalities, including scenario and driver metadata, natural language descriptions of the current scene and task, in-cabin camera images, driver operations, and information about surrounding vehicles.

The metadata includes information such as the data collection time, driver identity, driver condition, scenario category, and estimated task difficulty. It served as the key supervision for multi-label contrastive learning in MLLM as previously described in Section 3.3, and the rank learning and task difficulty estimation of the task–capability comparator as mentioned in Section 3.4.

Each segment of categorized scenarios is accompanied by a set of natural language descriptions to summarize the traffic context and the expected behavior of the ego vehicle. For example, in the scenario “IA2” shown in Figure 8a, one of the descriptions is “Stay in the left-turn lane and make a left at this urban junction. Ensure a safe gap from the vehicle in front.” Each set contains five semantically similar descriptions, from which one is randomly sampled when loading a data instance.

**Figure 9:**
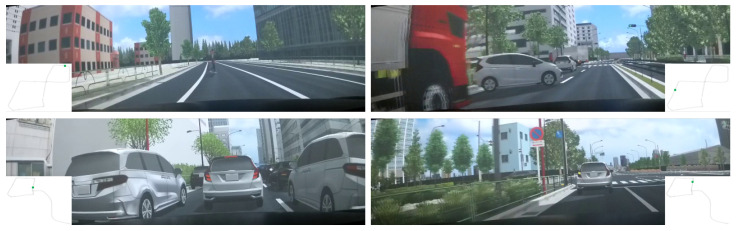
Examples of the in-cabin camera image modality. Reproduced from [38], distributed under the terms of the Creative Commons Attribution License (CC BY 4.0).

In-cabin camera images are captured at a resolution of 1920×1080 pixels and sampling rate of 30 Frames Per Second (FPS). The raw video data are downsampled to 2 FPS, resulting in 20 images per data sample. Each image is cropped to remove irrelevant or occluded regions and then resized to a resolution of 672×224 pixels, as shown in Figure 9. Subsequently, the images are normalized using CLIP [63] parameters and converted into tensors.

**Figure 10:**
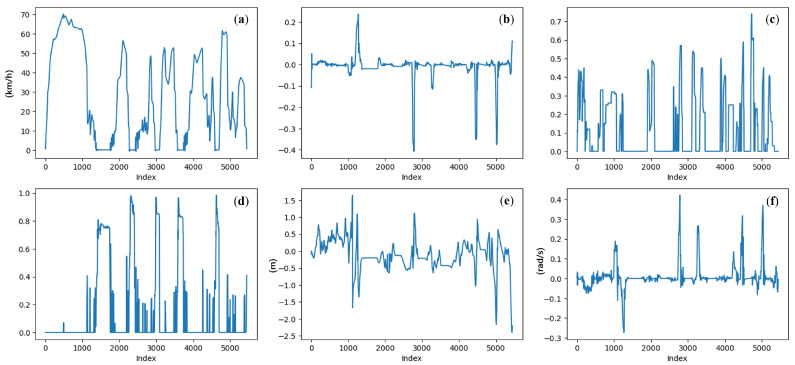
Example of the driver operation modality: (**a**) vehicle velocity (km/h); (**b**) steering angle ([−1, 1]); (**c**) throttle position ([0, 1]); (**d**) brake position ([0, 1]); (**e**) lane center deviation (m); (**f**) yaw rotation speed (rad/s). Reproduced from [38], distributed under the terms of the Creative Commons Attribution License (CC BY 4.0).

Driver operation data are collected from the simulator’s Controller Area Network (CAN) bus. In line with previous studies [38,39], we use six features to characterize the motion state of the ego vehicle and the driver’s manipulations, as shown in Figure 10. The data are downsampled to 10 frames per second and standardized using z-score normalization.

For information on surrounding vehicles, a set with a maximum length of 11 is constructed following the guidelines proposed in [80]. Each element in the set represents a surrounding vehicle and is described using five features: relative longitudinal and lateral positions, relative yaw angle, the surrounding vehicle’s speed, and the ego vehicle’s speed. As with driver operation data, surrounding vehicle information is also obtained directly from the simulator, downsampled to 10 FPS, and normalized using z-score normalization.

## 5. Experimental Evaluation

### 5.1. Experiment Setup

In the experiment, the whole framework is implemented with PyTorch [81] (Version 2.7.0). The task–capability large model is implemented on the LAVIS [82] (commit 506965b, accessed 7 May 2025) framework, while the visual encoder, Large Language Model (LLM), and the corresponding text tokenizer are realized using the transformers [83] library (Version 4.33.2). The visual encoder is based on CLIP [63], using the publicly released ViT-B/32 checkpoint (Hugging Face Hub ID: openai/clip-vit-base-patch32, accessed on 7 May 2025). The LLM and the corresponding text tokenizer are initialized with the publicly released LLaVA-v1.6-Vicuna-7B model (Hugging Face Hub ID: liuhaotian/llava-v1.6-vicuna-7b, accessed on 7 May 2025) from previous research [84].

Following previous studies [38,39], we partition the entire dataset into training, validation, and test sets with a ratio of 7:2:1 at the driving record level. The entire framework was trained in two stages:

**Stage 1: task–capability large model.** In the implementation, we treated the task-demand pipeline and the driving capability pipeline as two individual models. They share the same inputs except for the prompt, which are detailed in the Appendix A. For multi-label supervised contrastive learning, the capability pipeline uses status, category, and driver as supervisory labels with respective weights of 1, 0.5, and 0.25 to differentiate driving capabilities across different statuses, scenarios, and drivers. The demand pipeline, in contrast, employs category as its sole label. The hyperparameter settings for training were determined in accordance with BLIP-2 [10]. Both capability and demand pipelines were trained on the training set for 1000 epochs using the AdamW optimizer, with the standard “linear warm-up → cosine annealing” schedule. The learning rate started from $2\times 10^{-7}$, warming for 1000 steps to reach $2\times 10^{-5}$. The cosine decay progressively reduced the learning rate to $2\times 10^{-6}$. This training stage was executed in parallel on four NVIDIA RTX A6000 GPUs (NVIDIA Corporation, Santa Clara, CA, USA), each handling a batch of 16 samples.

**Stage 2: embedding comparator.** Since task difficulty lacks a precise quantitative definition, we adopt the Anomalous Proportion (AnoP) metric introduced in previous work [39] as the supervisory signal during training. AnoP represents the proportion of anomalous behavior within a given time window, and we posit that it is positively correlated with task difficulty. For the learnable function $g_{\mathrm{score}}\left(\cdot \right)$, the initial values of α and β are set to 0.01 and 5 to fit the distribution of AnoP. The embedding comparator was also trained on the training set for 1000 epochs, using Adam as the optimizer. The initial learning rate was set to $8\times 10^{-4}$ with a decay rate of 0.95 every 250 epochs. This stage was performed on a single NVIDIA RTX A6000 GPU.

### 5.2. Overall Evaluation Results

To evaluate whether the proposed framework produces meaningful representations of task demand d and driving capability c, we examined its performance on the test set and verified compliance with the properties defined in Section 3.1. The remainder of this section presents the experimental results in detail.

#### 5.2.1. Task Consistency

**Figure 11:**
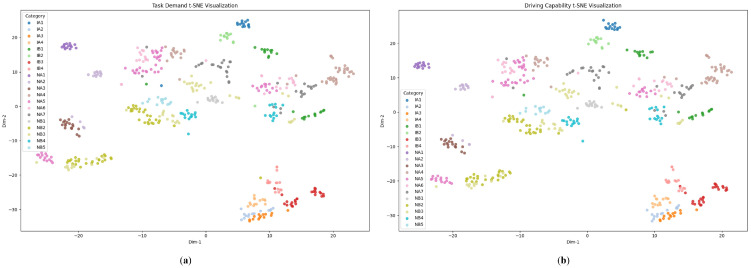
Scenario-wise t-SNE visualization of task demand and driving capability: (**a**) task demand; (**b**) driving capability.

In the proposed framework, an MLLM-based task–capability large model is employed to learn $f_{d}\left(\cdot \right)$ and $f_{c}\left(\cdot \right)$. The model extracts the task demand d and the driver’s delivered driving capability c from the driving environment and the driving behavior inputs, representing each as a 16-dimensional feature vector. As noted in Property 1 in Section 3.1, d and c should exhibit task consistency, i.e., the distance between d or c in similar scenarios should be smaller than that in dissimilar scenarios. To validate it, we evaluated the embeddings obtained from the test set. For each driving trip, we compute the average d and c for every scenario. The embeddings are further projected onto a two-dimensional plane using t-SNE, as shown in Figure 11. In the figure, each point represents one trip–scenario instance, and different colors correspond to different scenario categories.

**Figure 12:**
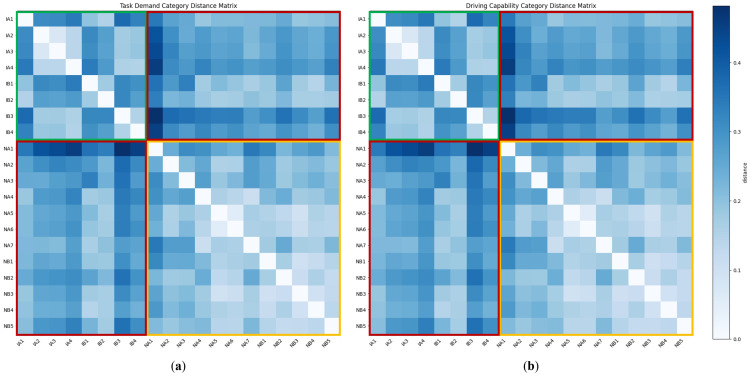
Inter-scenario Euclidean distance matrices of task demand and driving capability: (**a**) task demand; (**b**) driving capability. The colored frames highlight three region types: green—distances within intersection scenarios; yellow—distances within non-intersection scenarios; red—distances between intersection and non-intersection scenarios.

In Figure 11, it is observed that after the projection of t-SNE [85], the embeddings corresponding to different scenario categories are clearly separated, indicating that the model learns task-dependent demand and capability representations that are consistent within each scenario type. Moreover, the spatial pattern of task-demand embeddings in Figure 11a closely mirrors the pattern of driving capability embeddings in Figure 12b. Since drivers always attempt to allocate only the most appropriate capability level to the current driving context, the similarity observed here also suggests that the task–capability large model has learned demand and capability embeddings that sit in nearly the same latent space. To further quantify the inter-scenario separation, we compute the Euclidean distances between the centroids of all category clusters and visualize the results as a distance matrix as shown in Figure 12.

The distance matrix in Figure 12 is divided into three regions: green, yellow, and red. Overall, the green and yellow cells are visibly lighter than those in the red region, indicating that inter-class embedding distances (green/yellow) are larger than intra-class distances (red). Table 1 lists the mean distances for each region in detail. Since demand and capability cluster in a similar way, their distance statistics also line up closely. In particular, the distances between embeddings of the same scenario type are markedly larger than those between embeddings of different types, with values ranging roughly from 0.05 to 0.07. Within the same scenario class, the average distance for non-intersection scenes is lower than that for intersection scenes, with values around 0.02, suggesting that intersections are inherently more complex and variable, which is consistent with human intuition.

Table 2 dives deeper, listing how far the embedding of each scenario lies from the embeddings of a similar/different scenario group. Overall, the average distance between each scenario and similar scenarios exceeds that of the dissimilar ones, thereby satisfying Property 1. Figure 12 and Table 2 reveal that several scenes (IA1, IB1, IB2) exhibit embedding distances to scenarios of the same type that are nearly as large as their distances to scenes of different types. A common feature of these scenes is the absence of other vehicles or pedestrians. Under such circumstances, the intersection behaves more like a non-intersection curve than a car-following or pedestrian-containing intersection.

#### 5.2.2. Intra-Task Capability Ordering

**Figure 13:**
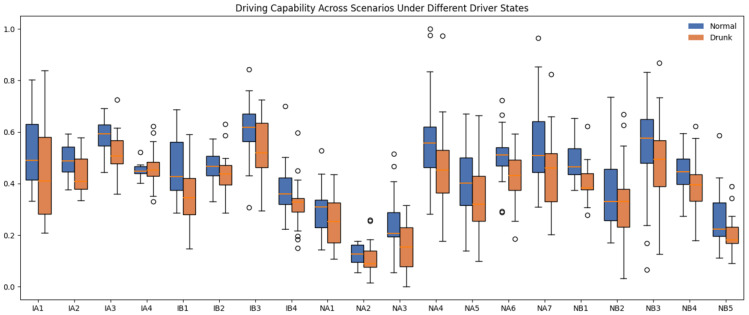
Distribution of normalized driving capability scores for normal and drunk drivers across different scenarios.

After generating the embedding of the driving capability c, the framework employs an embedding comparator to map it to a scalar space, resulting in a capability score $c_{\mathrm{score}}$. In this work, we introduce the Assumption 1, which states that a driver in a drunk state invariably exhibits lower capability than in the normal state. Experiments are adopted on the test set to verify whether the results comply with Property 2. Figure 13 illustrates the distribution of the driving capability scores under different driver states across all driving scenarios. A z-score normalization is applied to linearly rescale the values to the range [0,1].

In Figure 13, the blue boxes correspond to the normal state and the orange boxes to the drunk state. In almost every scenario, the drunk distribution shifts downward, resulting in systematically lower medians than the normal state, which supports Assumption 1 and satisfies Property 2 that impaired drivers exhibit reduced capability within the same task context. To further our analysis, we paired each driver’s driving capability scores before and after alcohol consumption within the same scenario and calculated the post-drinking reduction Δ. The score differences were classified into five categories: marked decrease (Δ≥0.1), slight decrease (0.05≤Δ<0.1), almost no change (−0.05<Δ<0.05), slight increase (−0.1<Δ≤−0.05), and marked increase (Δ≤−0.1). For each scenario, we calculated the proportion of pairs that fall into each category. The results are summarized in Table 3.

In general, only about 17% of the cases show an increase in the driving capability score of at least 0.05, while 54% exhibit a decrease of that magnitude, and the remaining 28% stay roughly unchanged. The intersection scenarios are the most vulnerable, where the “marked decrease” shares reach 50% in IA2, IA3, and all IB scenarios. In contrast, NA2, NA4, and several NB scenes have loss rates below 25% and high proportions of “almost no change”, suggesting that non-interaction road segments leave less room for model-detectable degradation. Figure 13 and Table 3 confirm that the degradation of driving capability after drinking dominates across scenarios, especially at intersections, reinforcing Property 2 of the study.

#### 5.2.3. Task–Capability Comparability

After obtaining the task-demand score and the driving capability score, the embedding comparator uses the learned $g_{\mathrm{score}}\left(\cdot \right)$ function to predict task difficulty. In our experiments, we fitted $g_{\mathrm{score}}\left(\cdot \right)$ to obtain task difficulty using AnoP, an objective metric proposed in previous work [38], as a ground-truth reference. Experiments are adopted in the test set to verify whether the results comply with the Property 3. The results are summarized in Table 4.

Table 4 indicates that the predicted task difficulty is consistently lower than the reference value in most cases and exhibits a relatively large mean absolute error (MAE) in several scenarios. However, the predicted difficulty for the drunk driving condition remains higher than that for the normal condition, which aligns with our expectations and gives the results a measure of practical significance. In general, the latent demand–capability representations can indeed be mapped onto a common, interpretable axis. However, the current results are suboptimal. Given that the other properties are well satisfied, the discrepancy may arise from factors such as: (i) the functional form of $g_{\mathrm{score}}\left(\cdot \right)$ may be inadequate to capture the true demand–capability–difficulty relationship; (ii) the reference metric may not coincide perfectly with the definition of task difficulty adopted in the TCI framework; or (iii) information may be lost when high-dimensional embeddings are compressed into scalar scores. In the following Section 5.3, we will investigate these possibilities in greater depth.

### 5.3. Ablation Study

To gain a deeper understanding of the proposed framework, we conduct a series of ablation experiments on different aspects. The results are detailed in the following sections.

#### 5.3.1. Embedding Dimension

In the experiments of Section 5.2, the embedding dimensionality of task demand and driving capability was set to 16. To further investigate how this hyperparameter influences the learned demand and capability representations, we additionally evaluate two comparison settings with 8 and 32 dimensions. All other hyperparameters are kept identical across these runs.

**Figure 14:**
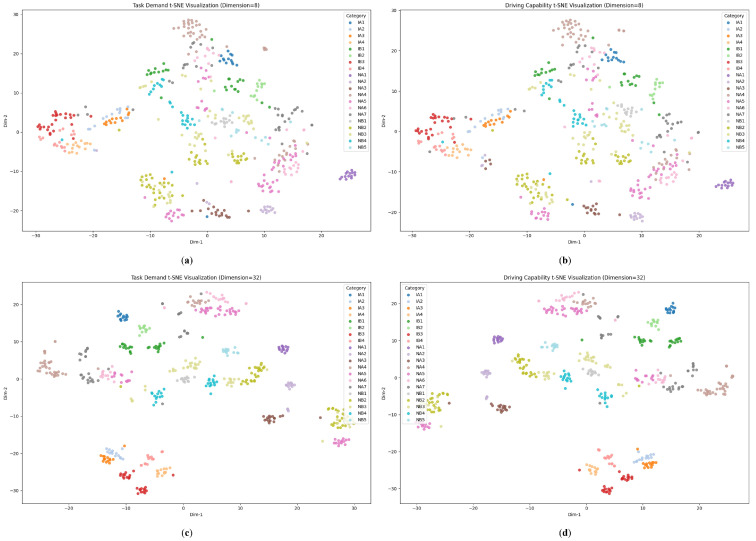
Impact of embedding dimensionality on cluster separability in the learned latent spaces: (**a**) task demand (dimension = 8); (**b**) driving capability (dimension = 8); (**c**) task demand (dimension = 32); (**d**) driving capability (dimension = 32).

Following the same procedure in Section 5.2.1, we visualize the learned embeddings using t-SNE as shown in Figure 14. Figure 14a,b show the 8-dimensional embeddings of the task demand and driving capability embeddings, respectively, Figure 14c,d depict the corresponding 32-dimensional embeddings. Comparison of the two plots shows that the t-SNE clusters are markedly more separable at 32 dimensions, whereas at 8 dimensions the clusters overlap heavily.

Similarly, the distances between scenarios of the same category and those of different categories are calculated. The results are summarized in Table 5. Increasing the latent dimension from 8 to 32 raises both intra-class and inter-class distances for demand and capability alike, which means that the clusters become more distinct; however, their internal cohesion also weakens. Currently, the dimension of 16 offers a balanced compromise. However, this is largely because our current scenario taxonomy is not highly fine-grained. We anticipate that once the scenarios are categorized at a more detailed level, a higher-dimensional embedding will yield better performance.

Table 6 presents the effect of the dimension of embedding of driving capability on the distribution of the reduction of the post-drinking driving capability score. As the embedding dimension increases, the ratio of the driving capability score reduction also increases, with the improvement particularly pronounced when the dimension increases from 8 to 16. However, when the dimensionality is further increased to 32, only marginal additional gain is offered, and the rate of “marked increase” also increases. In general, increasing embedding dimensionality enhances the sensitivity of the framework to capability degradation, but with tapering benefits beyond 16 dimensions under the current level of scenario granularity.

Table 7 summarizes how varying the dimensionality of demand and capability embedding spaces affects the accuracy of task-difficulty estimation. Although higher-dimensional embeddings can, in principle, encode richer information, the MAE remains nearly constant across the tested dimensions. We attribute this performance plateau to the final projection step: both embeddings are reduced to a single scalar before being passed to $g_{\mathrm{score}}\left(\cdot \right)$, creating a bottleneck that discards much of the structure that distinguishes one driving episode from another. Together with the findings in Section 5.2.3, these results suggest that compressing demand and capability to a single scalar and modeling $g_{\mathrm{score}}\left(\cdot \right)$ with a simple functional form is overly restrictive. A more expressive scoring mechanism, such as a small neural network operating on the full vector-valued embeddings, might preserve more latent information and may yield more accurate task-difficulty estimates. We leave this exploration in the following sections.

#### 5.3.2. Task-Difficulty Reference

As mentioned in Section 5.2.3, we fitted $g_{\mathrm{score}}\left(\cdot \right)$ to obtain task difficulty using AnoP as a reference to ground truth. In our previous experiments, we adopted the AnoP computed with the high-tolerance configuration reported in earlier work [38]. Consequently, its values clustered near 0, which may have hindered the ability of our function $g_{\mathrm{score}}\left(\cdot \right)=\log\left(1+\alpha e^{\beta (d_{\mathrm{score}}-c_{\mathrm{score}})}\right)$ to achieve a good fit. Consequently, we also include the reference obtained under a lower tolerance configuration in both training and evaluation, and investigate how this choice affects the fitting of $g_{\mathrm{score}}\left(\cdot \right)$. The results are presented in Table 8.

The results show that replacing the training reference with a stricter tolerance setting broadens the spread of the predicted task-difficulty scores. Under the stringent-tolerance reference, the ME even becomes positive, indicating that the model now overestimates the difficulty relative to the reference. However, the MAE remains comparatively large, reflecting limited accuracy. Employing both relaxed- and stringent-tolerance references in a joint-training scheme likewise widens the predicted range but fails to yield any meaningful reduction in MAE. In short, while the choice of task-difficulty reference metric clearly influences the numerical results, it is not the main cause of the suboptimal performance of the model. The following section investigates alternative explanations in more depth.

#### 5.3.3. Task-Difficulty Function

Considering that neither embedding dimensionality nor the choice of difficulty reference appears to be the decisive factor behind the suboptimal results, we next investigate the influence of the $g_{\mathrm{score}}$ function itself. Specifically, we replace the previous parametric formulation with a four-layer MLP and, in an extended setting, provide the raw demand and capability embeddings as additional inputs to the MLP. The corresponding results are summarized in Table 9.

In particular, simply replacing the original $g_{\mathrm{score}}$ function with an MLP actually degrades the accuracy. This decline may arise from low-dimensional input, which makes it difficult for the MLP to approximate the desired nonlinear mapping. However, the adoption of an MLP allows the embeddings of task demand and driving capability to be incorporated as part of the input. In this configuration, the MAE is significantly reduced, with the stringent-tolerance AnoP reference; the error is cut by about half compared with the baseline. The experimental results indicate that, compared to scalar scores, embeddings capture a more comprehensive set of information and, therefore, achieve higher accuracy in predicting task difficulty.

### 5.4. Generalization to Real-World Data

As noted in Section 4.1, our framework requires a dataset that contains multiple instances of driving behavior under similar tasks, with drivers exhibiting different levels of capability in those instances, in order to produce meaningful experimental results. To this end, we select the UAH-Driveset [86] to test the performance of our framework on real-world data. UAH-Driveset comprises six different drivers, two routes (motorway, secondary), and three distinct driving states (normal, drowsy, aggressive). The dataset offers multiple modalities that characterize both the driving environment and driver behavior, including in-car camera video and accelerometer signals.

For our experiments, we select the motorway route and extract only the car-following segments to align with our own data. The data format is also formed to fit our data format. For the various modalities, we proceed as follows:

- Image: Frames are sampled from the in-car camera footage.
- Description: A set of natural language descriptions is constructed to depict the car-following scenario; one sentence is randomly selected for each sample.
- Surroundings: Only the vehicle information directly ahead is used.
- Behavior: Because the angle of the steering wheel and the pedal positions are unavailable, we represent driver behavior using only vehicle speed, yaw rotation speed, and lateral deviation from the center of the lane.
- Prompt: The prompt is kept identical to that in our original dataset.

Since real-world traffic is highly dynamic, it is impossible to pair similar driving scenarios to what we have done through a simulator. To evaluate the proposed framework, we therefore calculate, for every sample, the ratio of the capability score to the demand score *r*, which reflects the moment-by-moment level of driver involvement. The experimental results are presented in Table 10.

As shown in Table 10, almost half of the normal samples lie in the balanced zone (0.95≤r≤1.05), with only 11.0% indicating a capability shortfall. When drivers become drowsy, the shortfall share almost doubles to 17.7%, while the proportion of marked surpluses (r≥1.10) drops to 8.6%, revealing a clear left shift caused by fatigue. In contrast, aggressive driving produces the opposite pattern: balanced episodes fall to 37.2%, whereas capability surpluses rise to 51.3%, reflecting frequent over-control associated with risk-taking behavior.

## 6. Discussion

This work takes an initial step toward the overarching goal of quantitatively modeling the Task–Capability Interface (TCI) so that misalignments between task demand and driving capability can be detected early and addressed in time to maintain safety. We have proposed a Multimodal Large Language Model (MLLM) framework that explicitly disentangles and quantifies the two TCI components. First, MLLM performs representation learning to obtain latent embeddings for task demand and driving capability. These embeddings are then projected into a common, orderable scalar space, enabling two complementary comparisons: the instantaneous gap between demand and capability, which flags potential risk moments; and the relative capability levels exhibited when the same task is performed by different drivers or by the same driver at different moments, providing a dynamic view of capability fluctuations. The experiments confirm that the learned representations satisfy the conceptual constraints introduced in Section 3.1: Property 1 and Property 2 are satisfied, while Property 3 is only partially met. Property 1 (task consistency) and Property 2 (intra-task capability ordering) are well met, as demonstrated by the larger inter-scenario embedding distances compared with intra-scenario distances and the monotonic degradation patterns observed after alcohol consumption. Property 3 (task–capability comparability) is partially satisfied: Although predicted difficulty preserves expected ordering between normal and impaired conditions, the mean absolute error (MAE) remains non-negligible. Finally, the framework generalizes to external data. In the subset of UAH-Driveset motorways, we observed clear state-dependent patterns: the capability-to-demand ratio shifted left under fatigue, indicating larger demand–capability gaps; and shifted right during aggressive maneuvers, reflecting the deployment of surplus capability. These trends align with established traffic psychology findings and underscore their interpretability.

Despite the encouraging results, there are still issues that remain open and deserve to be addressed. The first is a reliable estimate of task difficulty. Although the framework yields meaningful demand and capability embeddings, predicting task difficulty remains a challenge. Our experiments suggest that information loss in the embedding-to-scalar projection, together with the limited expressiveness of the current mapping function, contributes to this issue. However, a more fundamental obstacle is the lack of a precise and quantitative definition of task difficulty. AnoP used in the experiments might not be a perfect metric for task difficulty, and when alternative metrics are adopted, there is still a noticeable gap. It is necessary in the future to establish a reliable definition or representation of task difficulty. Another issue is establishing cross-task comparability. Because task demands are not yet comparable between heterogeneous driving tasks, capability comparisons can be performed only within similar tasks. Establishing cross-task comparability would “connect the dots,” linking isolated task-specific subgraphs into a unified demand–capability graph.

Building on this work, a practical and meaningful future direction is to generate natural language explanations alongside the extraction of task demand, driving capability, and task difficulty. Currently, in the proposed framework, the LLM is used to produce hidden states that are converted into demand and capability embeddings through a custom de-tokenizer. This procedure leverages the multimodal fusion of MLLM and the extensive latent knowledge in the LLM. However, an additional advantage of LLMs lies in their ability to generate coherent text. If the model could simultaneously output explanatory sentences, the resulting interpretations would enable more reasonable and situationally appropriate interventions.

## 7. Conclusions

This study proposed a data-driven route to operationalize the Task–Capability Interface (TCI) model by unifying visual, behavioral, and textual signals within a customized BLIP-2 architecture. The resulting low-dimensional embeddings preserve scenario semantics across diverse urban situations while capturing moment-to-moment capability variations induced by driver impairment. Empirically, the embeddings clustered tightly within similar tasks, the capability score consistently declined after alcohol consumption, and the scalar comparator reproduced the expected ordering between normal and impaired states, though residual errors suggest that a richer mapping could further improve accuracy. The framework also generalized to the real-world UAH Driveset without additional finetuning, revealing sensible capability—demand ratios during drowsy and aggressive maneuvers. In general, the proposed MLLM framework constitutes a promising step toward an explainable proactive risk assessment in intelligent vehicles, bridging the conceptual gap between task demand and driving capability and bringing us closer to co-drivers who can anticipate hazards before operational limits are exceeded.

## Figures and Tables

**Table 1:** Average Euclidean distance between latent embeddings of intersection and non-intersection scenario groups.

	I2I ↓	N2N ↓	I2N ↑
Demand	0.207	0.188	0.260
Capability	0.209	0.190	0.263

I2I = Intersection-to-Intersection; N2N = Non-intersection-to-Non-intersection; I2N = Intersection-to-Non-intersection. ↑ larger value signifies better results. ↓ smaller value signifies better results.

**Table 2:** Scenario-level Euclidean distances to intersection and non-intersection scenario groups.

Scenario	Demand			Capability		
	**Similar ↓**	**Different ↑**	**Average**	**Similar ↓**	**Different ↑**	**Average**
IA1	0.238	0.233	0.234	0.241	0.236	0.238
IA2	0.180	0.265	0.236	0.181	0.267	0.237
IA3	0.180	0.272	0.240	0.182	0.276	0.243
IA4	0.197	0.293	0.240	0.201	0.301	0.266
IB1	0.233	0.228	0.230	0.235	0.231	0.233
IB2	0.207	0.203	0.205	0.209	0.204	0.206
IB3	0.213	0.323	0.284	0.215	0.329	0.289
IB4	0.223	0.270	0.254	0.225	0.274	0.256
NA1	0.261	0.398	0.309	0.263	0.402	0.312
NA2	0.200	0.293	0.233	0.204	0.302	0.238
NA3	0.215	0.278	0.237	0.217	0.280	0.239
NA4	0.196	0.251	0.215	0.197	0.255	0.217
NA5	0.146	0.243	0.180	0.148	0.247	0.183
NA6	0.149	0.245	0.183	0.151	0.249	0.185
NA7	0.194	0.219	0.203	0.195	0.222	0.205
NB1	0.174	0.236	0.196	0.177	0.241	0.199
NB2	0.167	0.278	0.206	0.168	0.282	0.208
NB3	0.145	0.232	0.175	0.146	0.234	0.177
NB4	0.161	0.216	0.180	0.162	0.219	0.182
NB5	0.164	0.265	0.199	0.165	0.267	0.201

↑ larger value signifies better results. ↓ smaller value signifies better results.

**Table 3:** Scenario-wise distribution (%) of post-drinking driving capability score reduction.

Scenario	(−∞,−0.1] ↓	(−0.1,−0.05] ↓	(−0.05,0.05)	[0.05,0.1) ↑	[0.1,∞) ↑
IA1	16.7	0	16.7	16.7	50.0
IA2	5.6	0	33.3	33.3	27.8
IA3	5.6	11.1	27.8	5.6	50.0
IA4	16.7	11.1	61.1	5.6	5.6
IB1	9.1	3.0	21.2	12.1	54.5
IB2	11.8	17.6	23.5	17.6	29.4
IB3	11.8	2.9	23.5	2.9	58.8
IB4	10.5	5.2	31.6	10.5	42.1
NA1	5.6	11.1	38.9	16.7	27.8
NA2	10.0	10.0	60.0	15.0	5.0
NA3	5.3	5.3	21.1	26.3	42.1
NA4	6.6	9.8	21.3	13.1	49.2
NA5	9.7	8.3	23.6	20.8	37.5
NA6	8.3	2.8	16.7	30.6	41.7
NA7	15.1	12.1	27.2	9.1	36.3
NB1	0	5.9	47.1	5.9	41.2
NB2	8.1	12.9	37.1	12.9	29.0
NB3	15.7	2.0	13.7	23.5	45.1
NB4	5.7	14.3	31.4	17.1	31.4
NB5	5.9	5.9	47.1	11.8	29.4
Overall	9.4	7.8	28.4	15.9	38.5

↑ larger value signifies better results. ↓ smaller value signifies better results.

**Table 4:** Scenario-wise accuracy of the learned task-difficulty estimator.

Scenario	Normal			Drunk		
	**M.Ref**	**M.Pred**	**MAE ↓**	**M.Ref**	**M.Pred**	**MAE ↓**
IA1	0.068	0.002	0.065	0.130	0.080	0.100
IA2	0.072	0.034	0.041	0.113	0.081	0.054
IA3	0.038	0.011	0.034	0.031	0.074	0.063
IA4	0.068	0.016	0.054	0.101	0.022	0.090
IB1	0.070	0.032	0.050	0.161	0.150	0.104
IB2	0.042	0.006	0.039	0.098	0.007	0.092
IB3	0.051	0.014	0.044	0.096	0.031	0.076
IB4	0.042	0.013	0.031	0.052	0.025	0.040
NA1	0	0.001	0.001	0.003	0.001	0.002
NA2	0.002	0.006	0.006	0.005	0.027	0.025
NA3	0.041	0.022	0.036	0.090	0.126	0.124
NA4	0.015	0.002	0.014	0.042	0.017	0.026
NA5	0.013	0.001	0.013	0.033	0.005	0.032
NA6	0.025	0.002	0.023	0.046	0.018	0.041
NA7	0.006	0.003	0.006	0.068	0.032	0.046
NB1	0.007	0	0.007	0.006	0.002	0.006
NB2	0.008	0.001	0.008	0.023	0.007	0.018
NB3	0.008	0.002	0.009	0.019	0.005	0.017
NB4	0.021	0.006	0.017	0.042	0.032	0.045
NB5	0.043	0.039	0.052	0.054	0.059	0.061
Overall	0.029	0.008	0.026	0.055	0.031	0.045

M.Ref = mean reference task difficulty; M.Pred = mean predicted task difficulty; MAE = mean absolute error. ↓ smaller value signifies better results.

**Table 5:** Effect of embedding dimensionality on intra- and inter-category centroid distances.

Dimension	Demand		Capability	
	**Similar ↓**	**Different ↑**	**Similar ↓**	**Different ↑**
8	0.142	0.177	0.177	0.181
16	0.209	0.260	0.194	0.264
32	0.270	0.349	0.267	0.344

↑ larger value signifies better results. ↓ smaller value signifies better results.

**Table 6:** Effect of embedding dimensionality on the distribution (%) of post-drinking driving capability score reduction.

Dimension	(−∞,−0.1] ↓	(−0.1,−0.05] ↓	(−0.05,0.05)	[0.05,0.1) ↑	[0.1,∞) ↑
8	9.7	10.2	34.9	18.3	26.8
16	9.4	7.8	28.4	15.9	38.5
32	11.4	8.6	24.8	15.2	39.9

↑ larger value signifies better results. ↓ smaller value signifies better results.

**Table 7:** Effect of embedding dimensionality on the accuracy of task-difficulty estimation.

Dimension	Normal		Drunk	
	**ME**	**MAE ↓**	**ME**	**MAE ↓**
8	−0.015	0.025	−0.033	0.044
16	−0.022	0.026	−0.024	0.045
32	−0.025	0.027	−0.044	0.047

ME = mean error; MAE = mean absolute error. ↓ smaller value signifies better results.

**Table 8:** Effect of AnoP-tolerance configuration on the accuracy of the task-difficulty estimation.

Tolerance		Normal		Drunk	
**Relaxed**	**Stringent**	**ME**	**MAE ↓**	**ME**	**MAE ↓**
▲		−0.022	0.026	−0.024	0.045
	▲	0.055	0.110	−0.195	0.209
▲	Δ	−0.012	0.026	−0.037	0.047
Δ	▲	0.063	0.114	−0.178	0.196

ME = mean error; MAE = mean absolute error. ▲ means it is used in both training and evaluation; Δ means it is only used during training. ↓ smaller value signifies better results.

**Table 9:** Effect of task-difficulty function on the accuracy of the task-difficulty estimation.

Method	Tolerance		Normal		Drunk	
	**Relaxed**	**Stringent**	**ME**	**MAE ↓**	**ME**	**MAE ↓**
baseline	▲		−0.022	0.026	−0.024	0.045
w/ NN	▲		−0.019	0.027	−0.044	0.051
w/ NN & EMB	▲		−0.014	0.021	−0.016	0.037
baseline		▲	0.055	0.110	−0.195	0.209
w/ NN		▲	−0.011	0.083	−0.276	0.283
w/ NN & EMB		▲	−0.012	0.061	−0.063	0.102

ME = mean error; MAE = mean absolute error. NN means a neural network served as $g_{\mathrm{score}}\left(\cdot \right)$. EMB means using the embeddings of task demand and driving capability together with scores as the input of $g_{\mathrm{score}}\left(\cdot \right)$. ▲ means it is used in both training and evaluation. ↓ smaller value signifies better results.

**Table 10:** Distribution (%) of capability-to-demand score ratios under different driver states on the UAH-Driveset motorway subset.

Status	(0,0.9]	(0.9,0.95]	(0.95,1.05)	[1.05,1.1)	[1.1,1.2)	[1.2,∞)
Normal	4.8	6.2	48.6	23.1	10.6	6.7
Drowsy	6.9	10.8	46.9	26.8	5.3	3.3
Aggressive	5.1	6.4	37.2	34.1	9.4	7.8

## Data Availability

The dataset utilized in this project was collected from the driving simulator in the Advanced Research and Innovation Center, DENSO CORPORATION. This dataset has been specifically provided for the purpose of conducting various experiments within this project.

## Abbreviations

The following abbreviations are used in this manuscript:

WHO	World Health Organization
ABS	Anti-lock Braking Systems
ESC	Electronic Stability Control
ADAS	Advanced Driver-Assistance Systems
ACC	Adaptive Cruise Control
AEB	Autonomous Emergency Braking
LKA	Lane Keeping Assist
TCI	Task–Capability Interface
MLLM	Multimodal Large Language Model
SupCon	Supervised Contrastive learning
MLP	Multi-Layer Perceptron
FPS	Frames Per Second
CAN	Controller Area Network
LLM	Large Language Model
AnoP	Anomalous Proportion
MAE	Mean Absolute Error

## Appendix A. Prompt of the Task–Capability Large Model

In the task–capability large model, the prompt comprises two components. The first component provides a general description of the data; for instance, when we use the simulator dataset whose driving scenes are modeled after Japanese roadway, it is “The driving environment assumes left-side traffic, so turns, lane changes, and yield rules are consistent with left-hand driving.” And for the evaluation on the UAH-Driveset, which was recorded on Spanish roads, the General Description section of the prompt is adjusted to emphasize right-side traffic.

The second component specifies the task itself. For the task-demand pipeline, the prompts are as follows:

- Describe the overall task demand presented by the driving environment in this scene.
- Based on the input data, what are the primary environmental and situational challenges the driver faces during this period?
- Summarize the situational demands posed by this scene, including traffic, road structure, and potential hazards.
- Based on the provided information, estimate the level of attentional and control effort required to navigate this scene.
- How do the current traffic and road conditions impact the complexity of the driving task?

And the driving capability prompts are defined as follows:

- Describe the driving capability demonstrated by the driver in this scene.
- Evaluate the driver’s performance based on the scene and driving behavior provided.
- Based on the input data, what can you infer about the driver’s driving skill and behavior during this period?
- Given the visual and sensor data, extract indicators of driver control quality and risk awareness.
- Summarize the driver-delivered capability using the combined perception from image and sensor data.

Every time the model is called, one prompt configuration is randomly selected from the pool of available combinations and used alongside the current input for inference.
